# Early postoperative plasma circulating tumour DNA for molecular residue disease detection and recurrence risk evaluation in surgical non‐small cell lung cancer

**DOI:** 10.1002/ctm2.70056

**Published:** 2024-10-11

**Authors:** Jie Tang, Yingsong Tian, Song Wang, Yong Liu, Manjun Chen, Xudong Yang, Xinghe Tong, Mengtian Wang, Yunping Zhao, Jiaohui Pang, Qiuxiang Ou, Xiaobo Chen

**Affiliations:** ^1^ Department of Thoracic Surgery The First Affiliated Hospital of Kunming Medical University Kunming Yunnan China; ^2^ Geneseeq Research Institute, Nanjing Geneseeq Technology Inc. Nanjing Jiangsu China

Dear Editor,

This study emphasises the potential of utilising early postoperative plasma circulating tumour DNA (ctDNA) to predict tumour relapse in patients with resectable non‐small cell lung cancer (NSCLC) while considering region‐specific susceptibility within a real‐world study cohort.

Lung cancer remains the predominant cause of cancer‐associated mortality, with NSCLC constituting nearly 85% of cases. Standard treatment for operable NSCLC typically involves curative‐intent surgery, but the high risk of recurrence necessitates further exploration of effective biomarkers for monitoring and personalised treatment. Advances in detecting ultra‐low‐frequency somatic genomic alterations in plasma ctDNA have shown potential for identifying patients at higher risk of tumour relapse in NSCLC.^1^, ^2^ Nonetheless, the prognostic value of ctDNA‐based molecular residue disease (MRD), especially concerning regional variations in lung cancer risk, remains unexplored. Xuanwei, a city in Yunnan province, China, offers an opportunity to investigate genetic attributes influencing lung cancer due to its exceptionally high incidence linked to indoor air pollution from smoky coal combustion.^3^

Here, we retrospectively analysed targeted next‐generation sequencing data of primary tumours from 226 stage I–III patients with resectable NSCLC, including 31 from the Xuanwei region (Figure S1). Adenocarcinoma was the predominant histological subtype, and most patients presented with stage I disease (Table 1). Notably, Xuanwei patients exhibited a higher incidence of multifocal lesions and tumours with high TMB (Figure 1A–C). Frequently mutated genes included *EGFR* (62%), *TP53* (32%), *KRAS* (18%), and *LRP1B* (12%), while additional mutations were identified in oncogenes or tumour suppressor genes, such as *ALK*, *RB1*, *ERBB2*, *PIK3CA*, *BRAF*, *NTRK1*, and *ARID1A*. Furthermore, Xuanwei patients showed more uncommon *EGFR* mutations associated with US Food and Drug Administration (FDA)‐approved treatments (*p *< .001)^4^ and fewer common mutations like p.L858R and exon 19 deletions (19del), but higher p.G719X and p.S768I frequencies (Figure 1D and E). This distinct distribution of *EGFR* variants was confirmed using the MSKCC and OncoSG cohorts and was consistent with previous studies (Figure 1F).^5^ Gene enrichment analysis revealed six genes with higher mutational frequencies in Xuanwei, among which *KRAS* and *PIK3CA* mutations might provide insights due to their clinical actionability (Figure 1G). Here, we showed that KRAS p.G12C (51.1%, 23/45) and PIK3CA p.E545K (20%, 3/15) mutations were predominant, with mutations like KRAS p.G12A, p.Q61E, and PIK3CA p.G106V, p.P471H exclusive to Xuanwei patients (Figures 1H and I and S2).

**TABLE 1:** Clinical characteristics of patients.

Characteristic	Total (*N* = 226)	Xuanwei (*N* = 31)	Non‐Xuanwei (*N* = 195)	*p* Value
Sex				.44
Male	100 (44.2%)	16 (51.6%)	84 (43.1%)	
Female	126 (55.8%)	15 (48.4%)	111 (56.9%)	
Age at diagnosis				.85
Median (IQR)	56 (50–64)	54 (49–63)	57 (50–65)	
≥ 60 years	93 (41.2%)	12 (38.7%)	81 (41.5%)	
< 60 years	133 (58.8%)	19 (61.3%)	114 (58.5%)	
Histological subtype				.23
Adenocarcinoma	211 (93.4%)	31 (100.0%)	180 (92.3%)	
Non‐adenocarcinoma	15 (6.6%)	0 (.0%)	15 (7.7%)	
Clinical stage				.28
I	192 (85.0%)	24 (77.4%)	168 (86.2%)	
II‐III	34 (15.0%)	7 (22.6%)	27 (13.8%)	
Smoking history				.19
Yes	38 (16.8%)	8 (25.8%)	30 (15.4%)	
No	155 (68.6%)	19 (61.3%)	136 (69.7%)	
Unknown	33 (14.6%)	4 (12.9%)	29 (14.9%)	
Lesion				<.01
Multifocal	43 (19.0%)	12 (38.7%)	31 (15.9%)	
Singular	183 (81.0%)	19 (61.3%)	164 (84.1%)	
PD‐L1, TPS				.33
≥1%	42 (18.6%)	8 (25.8%)	34 (17.4%)	
< 1%	162 (71.7%)	21 (67.7%)	141 (72.3%)	
Unknown	22 (9.7%)	2 (6.5%)	20 (10.3%)	
TMB				<.001
≥10 mut/Mb	31 (13.7%)	14 (45.2%)	22 (11.3%)	
< 10 mut/Mb	119 (52.7%)	9 (29.0%)	128 (65.6%)	
Unknown	76 (33.6%)	8 (25.8%)	45 (23.1%)	
Therapy				<.01
Tumour resection	110 (48.7%)	9 (29.0%)	101 (51.8%)	
Adjuvant chemotherapy	57 (25.2%)	9 (29.0%)	48 (24.6%)	
Adjuvant targeted therapy	29 (12.8%)	3 (9.7%)	26 (13.3%)	
Adjuvant chemotherapy + targeted therapy	16 (7.1%)	7 (22.6%)	9 (4.6%)	
Others^a^	14 (6.2%)	3 (9.7%)	11 (5.6%)	

Abbreviations: IQR, interquartile range; PD‐L1, programmed death ligand 1; TPS, tumour proportion score.

^a^ Others refer to radiotherapy alone, immunotherapy alone, or any combination of chemotherapy, radiotherapy, and immunotherapy in the adjuvant setting.

**FIGURE 1:**
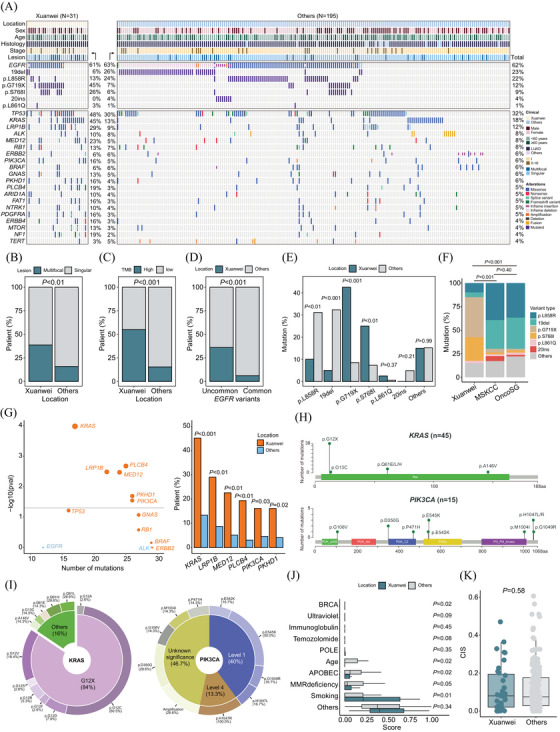
Genomic landscape of patients. (**A**) Heatmap showing baseline characteristics and somatic genomic variant profiling of patients. (**B–D**) Bar plots depicting the proportion of patients from Xuanwei or other regions within the country, stratified by primary lung cancer lesion (B), tumour mutational burden (C), and *EGFR* variants (D). (**E**) Detection rate of common (p.L858R and exon 19 deletion) and uncommon *EGFR* mutations (p.G719X, p.S768I, p.L861Q, and exon 20 insertions) among patients from Xuanwei and other regions. (**F**) External validation of *EGFR* variant type distribution using MSKCC and OncoSG cohorts. (**G**) Gene‐level enrichment of genetic variants with a mutational frequency of at least 5% in patients stratified by location. Colour represents the enrichment group, while dot size reflects Fisher's exact test *p*‐values. (**H**) Type and mutational site of *KRAS* and *PIK3CA* mutations identified within the study cohort. (**I**) Pie charts show the proportion of *KRAS* and *PIK3CA* variants, categorised by KRAS p.G12X mutations and the OncoKB‐defined level of clinical actionability, respectively. (**J**) Box plots showing the distribution of mutational signature scores for each patient. (**K**) Box plot comparing the chromosomal instability score (CIS) between patients from Xuanwei and other regions.

Mutational signature analysis revealed higher smoking scores but lower scores for age, apolipoprotein B mRNA editing catalytic polypeptide‐like (APOBEC), and DNA mismatch repair deficiency (dMMR) in Xuanwei patients (Figure 1J). Chromosomal instability score and survival outcomes, however, did not differ between patients from different regions (Figures 1K and S3). Indeed, Wang et al. found that the APOBEC mutational signature correlates with a sustained response to checkpoint blockade immunotherapy in NSCLC.^6^ Conversely, inactivation of the MMR pathway can lead to the accumulation of DNA replication errors and microsatellite instability, which is the first FDA‐approved tumour‐agnostic biomarker for immunotherapy.^7^ Collectively, these findings suggest that immunotherapy may have limited efficacy for Xuanwei patients. Despite this, the notably higher prevalence of atypical *EGFR* mutations, along with mutations in *KRAS* and *PIK3CA* highlights the potential for individualised precision medicine and underscores the need for developing targeted agents against these specific mutations.

The DYNAMIC study was the first prospective study to determine the appropriate time for MRD detection, demonstrating that ctDNA detection on the third day following radical resection is associated with shorter disease‐free survival (DFS).^1^ Moreover, ctDNA evaluation as early as 3 to 7 days postoperatively was significantly associated with an increased recurrence risk in colorectal cancer patients.^8^ In our study, plasma samples were collected within 3–7 days post‐surgery (median 4.5, interquartile range [IQR]: 4.0–6.0 days), to identify high‐risk patients for disease recurrence using a tumour‐informed MRD detection method (Supplementary Material). The interval allows for near‐complete clearance of ctDNA from surgical trauma, ensuring any detected ctDNA reflects residual disease. Additionally, this timing aligns with typical hospital stays, making it practical for timely MRD testing and treatment planning. Detectable ctDNA demonstrated a high specificity of .96 for predicting tumour relapse, with specificity reaching .97 in stage I patients and .73 in stage II–III patients (Figure 2A). The overall sensitivity was .67, with .38 for stage I and .83 for stages II–III. Twelve patients with undetectable ctDNA recurred, resulting in a negative predictive value (NPV) of .94, which further increased to .96 for early‐stage patients (Figure 2B). Survival analysis demonstrated strong associations between ctDNA MRD and survival outcomes, with ctDNA‐negative patients exhibiting substantially longer times to tumour relapse and improved overall survival (OS) (Figure 2C). Among 24 patients with post‐surgical detectable ctDNA, the median lead time to radiologically confirmed tumour relapse was 11.5 months (IQR: 6.0–19.0 months) (Figure 2D). Conversely, the median tumour relapse time was 14.5 months (IQR: 9.0–21.5 months) for ctDNA‐negative patients. Clinical and mutational features with a mutation frequency of ≥ 5% in the study cohort were further analysed using multivariate analysis if they were significantly associated with DFS and OS in univariate Cox proportional hazard models (Table S1, S2). Importantly, MRD status, clinical stage, and *RB1* variants were independently associated with DFS (Figure 3A). The prognostic value of ctDNA‐based MRD remained significant for stage‐matched patients and *RB1* wild‐type patients (Figure 3B–E). For overall survival, both age and clinical stage remained significant in multivariate analysis (Figure S4A). Detectable ctDNA effectively stratified high‐risk patients, particularly those over 60 years of age or with stage II‐III disease (Figure S4B–E). In Xuanwei patients, positive ctDNA detection also predicted tumour relapse but was not significantly associated with OS (Table S3).

**FIGURE 2:**
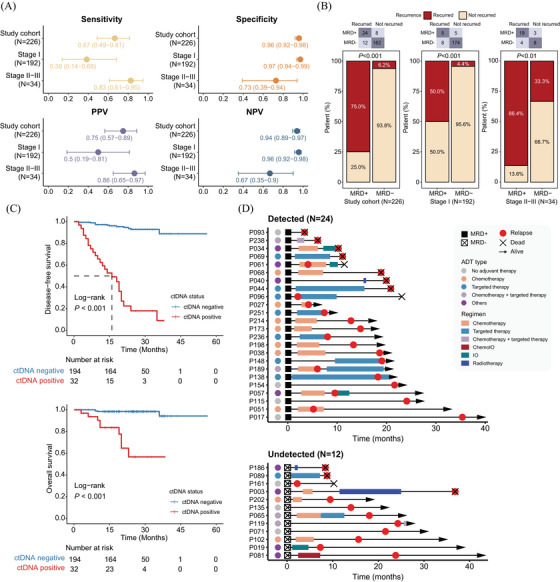
MRD detection in postoperative plasma samples predicts tumour recurrence. (**A**) The performance of MRD detection in plasma ctDNA for predicting tumour relapse within the study cohort, stage I patients, and stage II–III patients. (**B**) Bar plots showing the distribution of patients with or without disease recurrence stratified by MRD status. (**C**) Kaplan–Meier analysis of disease‐free survival and overall survival between patients with or without detectable ctDNA. (**D**) MRD assessment and treatment overview for all 36 recurred patients. MRD, molecular residue disease; PPV, positive predictive value; NPV, negative predictive value; ADT, adjuvant therapy; IO, immunotherapy; ChemoIO, chemo‐immunotherapy.

**FIGURE 3:**
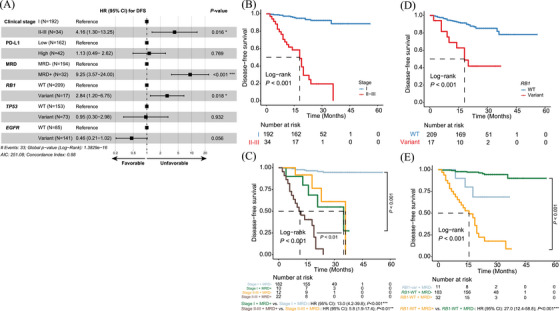
Detectable MRD associated with worse disease‐free survival in surgical patients. (**A**) Forest plot showing the multivariate analysis of hazard ratios (HR) with 95% confidence intervals (CI) for disease‐free survival (DFS). (**B, C**) Kaplan–Meier analysis of DFS stratified by clinical stage or in combination with postoperative molecular residual disease (MRD) status. (**D, E**) Kaplan–Meier analysis of DFS stratified by baseline *RB1* variants or in combination with postoperative MRD status.

Our study unveiled that MRD detection reliably predicts tumour relapse with high specificity but suboptimal sensitivity whether in early‐stage or locally advanced NSCLC patients. The low sensitivity could be attributed to several reasons, including the fact that most patients (85%) had stage I NSCLC with small tumours, which may hinder the detection of genomic variants using current sequencing technologies. Likewise, Zhong et al. observed a similar sensitivity of .41 (95% CI: .35–.46) for NSCLC patients with stage I‐III disease and .31 (95% CI: .24–.38) for those with stage I.^9^ Additionally, the histological subtype may also affect ctDNA detection rates, as demonstrated in the TRACERx study, which found a 19% positive detection rate in lung adenocarcinoma patients.^10^ Furthermore, some tumours may not shed ctDNA, especially for patients with brain‐only recurrences.^2^ Nonetheless, this does not seem to undermine the feasibility of ctDNA in detecting MRD for monitoring tumour relapse.

This study has several limitations. The limited sample size from the Xuanwei region may affect the robustness of statistical analysis, necessitating a larger cohort to validate the main findings. Additionally, while the inclusion of diverse risk profiles enhances the generalisability of our primary finding that ctDNA‐based MRD detection enables effective recurrence risk evaluation, a nested case‐control study could further reduce potential confounding in subgroup analyses by more precisely matching patients on key variables such as clinical stage and smoking history. Lastly, while the study highlighted the high specificity of MRD detection for lung cancer, results should be interpreted with caution due to the relatively low sensitivity, especially for patients with undetectable ctDNA.

## CONCLUSION

In conclusion, comprehensive genomic profiling revealed distinct clinicopathological features among patients with varying regional lung cancer risks. The prognostic value of postoperative ctDNA MRD was reinforced in a real‐world cohort of surgical NSCLC patients.

## AUTHOR CONTRIBUTIONS

**Jie Tang and Yingsong Tian**: conceptualisation; formal analysis; investigation; methodology; writing—original draft; writing – review & editing. **Song Wang**: formal analysis; investigation; writing – original draft; writing – review & editing. **yong liu**: formal analysis; investigation; writing – review & editing. **Manjun Chen**: investigation; writing – review & editing. **Xudong Yang**: investigation; funding acquisition; writing – review & editing. **Xinghe Tong; Mengtian Wang; Yunping Zhao**: investigation; writing – review & editing. **Jiaohui Pang**: methodology; writing – review & editing. **Qiuxiang Ou**: writing – review & editing. **Xiaobo Chen**: conceptualisation; investigation; resources; data curation; supervision; writing – review & editing.

## CONFLICT OF INTEREST STATEMENT

SW, YL, JHP, and QXO are employees of Nanjing Geneseeq Technology Inc. The remaining authors declare no competing interests.

## FUNDING INFORMATION

This study was supported by the Yunnan Provincial Department of Science and Technology Foundation for Youths (202301AU070195) and Scientific Research Foundation for Doctors, the First Affiliated Hospital of Kunming Medical University (2021BS017) to Xudong Yang. The funder had no role in the study design, data collection and analysis, or decision to publish the manuscript.

## ETHICS STATEMENT

The study was approved by the Ethics Committee of the First Affiliated Hospital of Kunming Medical University (approval number: (2022) ERL No. 196). Written informed consent was obtained from each patient before sample collection.

## Supporting information



Supporting information

## Data Availability

The datasets generated and/or analysed during this current study are available from the corresponding author upon reasonable request.
