# Supplementary material for: Early postoperative plasma circulating tumour DNA for molecular residue disease detection and recurrence risk evaluation in surgical non‐small cell lung cancer
Source: Clin Transl Med. 2024 Oct 11;14(10):e70056. doi: 10.1002/ctm2.70056 (PMC11469951; doi:10.1002/ctm2.70056)
Supplement: Supplementary file 1 — Supporting information [file CTM2-14-e70056-s001.docx]

**Supplementary Materials**

## **Materials and Methods**

### **Patients and samples**

Between July 2020 and December 2022, a total of 249 patients were histologically diagnosed with NSCLC at the First Affiliated Hospital of Kunming Medical University. The primary inclusion criteria of patients were as follows: i) patients who had not received any treatment before curative-intent tumor resection; ii) early-stage or locally advanced NSCLC patients (stage IA-IIIC) who underwent R0 resections confirmed by hematoxylin and eosin (H&E) staining and light microscopy techniques; iii) surgical tissue samples were sequenced on a lung cancer-specific next-generation sequencing (NGS) panel (Pulmocan^®^, Nanjing Geneseeq Technology Inc., Nanjing, China) or a pan-cancer panel (GeneseeqPrime^®^, Nanjing Geneseeq Technology Inc., Nanjing, China) for genomic alteration detection; iv) paired plasma samples at 3-7 days after surgery were available. Among all the 249 patients, 20 were deemed inoperable due to stage IV disease or had incomplete information on the clinical stage, while 3 patients had negative variant detection in their surgical samples. Consequently, these 23 patients were excluded from further analyses, leaving 226 patients with paired surgery-resected tumor tissues and plasma samples in the study cohort. A total of 43 patients were diagnosed with multifocal lung cancer based on the American College of Chest Physicians (ACCP) guidelines [1]. Among these, 14 patients had only one primary tumor included in further analysis, either due to sequencing failure or economic reasons. Ultimately, 262 surgical samples were analyzed, consisting of 183 samples from patients with single primary tumors and 79 samples from those with multifocal lesions. Data from two external datasets, hereafter referred to as MSKCC and OncoSG, were utilized to validate the distinct pattern of *EGFR* mutation subtypes, which was downloaded from cBioportal (https://www.cbioportal.org/) [2, 3]. This study was approved by the ethics committee of the First Affiliated Hospital of Kunming Medical University (ethical number: (2022) ERL No.196) and conducted in accordance with the principles of the Helsinki Declaration. All patients provided written informed consent to participate and for publication.

### **Library construction and sequencing**

Formalin-fixed paraffin-embedded (FFPE) samples underwent de-paraffinization with xylene, and genomic DNA extraction was conducted using the QIAamp DNA FFPE Tissue Kit (Qiagen Cat. No. 56404) following the manufacturer’s instructions. The plasma fraction of the peripheral blood was isolated within two hours after specimen collection, which was first centrifuged at 1,800 g for 10 min, followed by circulating cell-free DNA (cfDNA) extraction and purification using the QIAamp Circulating Nucleic Acid Kit (Qiagen Cat. No. 55114). Extraction of genomic DNA from white blood cells in sediments for each specimen was performed using the DNeasy Blood and Tissue Kit (Qiagen Cat. No. 69504), subsequently utilized as the normal control. Purified DNA was qualified and quantified using Nanodrop2000 (Thermo Fisher Scientific, Waltham, MA) and the dsDNA HS assay kit on a Qubit 3.0 fluorometer (Life Technology, US), respectively. NGS libraries were constructed using the KAPA Hyper Prep kit (KAPA Biosystems) with an optimized manufacturer’s protocol for different sample types. Hybridization-based target enrichment was carried out using xGen lockdown probes targeting 139 lung cancer-related genes (Pulmocan^®^, Nanjing Geneseeq Technology Inc., Nanjing, China) or 437 pan-cancer genes (GeneseeqPrime^®^, Nanjing Geneseeq Technology Inc., Nanjing, China). Fragment size distribution was determined using the Agilent Bioanalyzer 2100 system (Agilent Technologies, Santa Clara, USA). The target-enriched library was then sequenced on the Illumina Hiseq4000 platform according to the manufacturer’s instructions.

### **Somatic variant calling in baseline tumor samples**

For quality control, sequencing data underwent demultiplexing and subsequent low-quality reads trimming using Trimmomatic [4]. Qualified data were then aligned to the human reference genome (Hg19) with default parameters using the Burrows-Wheeler Aligner (BWA-mem, v0.7.12; <https://github.com/lh3/bwa/tree/master/bwakit>). The Genome Analysis Toolkit (GATK 3.4.0; <https://software.broadinstitute.org/gatk/>) was employed for recalibrating base quality score and for local realignment around insertions/deletions (indels). Duplicated reads were removed using Picard tools to enhance data accuracy. VarScan2 was used to identify single-nucleotide variations (SNVs) and indels. For primary tumors, a mutation was considered a candidate somatic mutation only when: i) it was not present in matched genomic DNA from white blood cells; ii) it was not present in >1% of the population in the 1000 Genomes Project or the Exome Aggregation Consortium 65,000 exomes database; iii) variant allele frequency (VAF) ≥0.5% and supporting reads ≥3 for hotspot mutations (≥20 mentions in COSMIC v92 [5]); VAF ≥1% and supporting reads ≥6 for non-hotspot mutations. Copy number variants (CNVs) were analyzed using copy number values adjusted by sample ploidy using FACETS [6], with a gene ratio cutoff of ≥2.0 for copy number gain and <0.6 for copy number loss. Gene fusions with split-reads ≥6 were reported by DELLY using default parameters [7]. All candidate variants were manually verified using the Integrative Genomics Viewer [8].

Tumor mutation burden (TMB) was calculated as the total number of somatic mutations and indels per megabyte bases in coding regions detected for each primary tumor tissue, including synonymous alterations and excluding known driver mutations as previously described [9]. For patients diagnosed with multiple primary lung cancer, where more than one tumor sample was sequenced, the TMB for each patient was determined by the highest TMB value calculated across samples [10]. A threshold of ≥10 mutations per megabase (muts/Mb) was used to classify patients as TMB-high (TMB-H), while those with <10 muts/Mb were classified as TMB-low (TMB-L). Chromosomal instability score (CIS) was defined as the average proportion of the genome with aberrant copy number (log2 depth ratio >±0.2), weighted on each of the 22 autosomal chromosomes [11].

### **Mutational signature analysis**

Samples with at least 5 synonymous/non-synonymous mutations were included in the mutation signature analysis [12], which was conducted using the “maftools” and “sigminer” R packages. The 30 mutational signatures outlined on the COSMIC website (https://cancer.sanger.ac.uk/signatures/signatures_v2/) were categorized into ten groups as previously described (**Table S4**) [13].

### **MRD detection of plasma ctDNA**

Variant calling of plasma samples was based on the tumor-informed method. After annotation, variants associated with clonal hematopoiesis were filtered out by comparing them to those detected in paired white blood cell samples. A variant was considered an eligible patient-specific somatic variant only if it met the following criteria: i) it was absent in matched genomic DNA from white blood cells; ii) it was not present in >1% of the population in the 1000 Genomes Project or the Exome Aggregation Consortium 65,000 exomes database; iii) it was identified as a candidate somatic variant in the matched primary tumor; iv) supporting reads ≥2 for mutations and indels; v) a gene ratio cutoff of ≥1.6 for copy number gain and <0.6 for copy number loss; vi) split-reads ≥2 for gene fusions. All candidate variants were manually verified using the Integrative Genomics Viewer. A plasma sample was defined as ctDNA-positive if at least one tumor-derived variant was detected.

### **PD-L1 expression analysis**

Immunohistochemistry (IHC) staining of programmed death ligand 1 (PD-L1) was conducted using the Dako PD-L1 IHC 22C3 pharmDx kit (Agilent Technologies) following the manufacturer’s instructions [14]. PD-L1 expression level was determined based on the tumor proportion score (TPS), calculated as the number of PD-L1-stained positive tumor cells divided by the total number of viable tumor cells multiplied by 100. A TPS value of ≥1% was considered indicative of PD-L1 positivity.

### **Statistical analysis**

All statistical analyses were performed in R (version 4.1.3). Fisher’s exact test was used to compare the frequencies of categorical variables across different groups, while the Wilcoxon rank sum test was employed to compare the distribution of continuous data. Using the epiR package, we computed the sensitivity, specificity, positive predictive value (PPV), and negative predictive value (NPV) based on true positives (TP), true negatives (TN), false positives (FP), and false negatives (FN) for MRD detection, with tumor recurrence data serving as the reference. Disease-free survival (DFS) was calculated as the duration from radical surgery to tumor relapses or patient death, while overall survival (OS) was determined based on the time from initial diagnosis to patient death. For patients lost to follow-up, OS was calculated based on the time of the last follow-up. Kaplan-Meier survival curves were used to compare the survival of subgroup patients, with statistical differences assessed using the log-rank test. Cox proportional hazard models were fitted to estimate hazard ratios (HRs) with 95% confidence intervals (CIs), and the proportionality of hazards was assessed using log(-log) survival plots. A two-sided *P*-value of less than 0.05 was considered significant for all tests unless otherwise indicated (**P*<0.05, ***P*<0.01, ****P*<0.001).

## **Supplementary References**

1. Kozower B. D., Larner J. M., Detterbeck F. C. and Jones D. R., *Special treatment issues in non-small cell lung cancer: Diagnosis and management of lung cancer, 3rd ed: American College of Chest Physicians evidence-based clinical practice guidelines.* Chest, 2013. **143**(5 Suppl): p. e369S-e399S.

2. Caso R., Sanchez-Vega F., Tan K. S., Mastrogiacomo B., Zhou J., Jones G. D., et al., *The Underlying Tumor Genomics of Predominant Histologic Subtypes in Lung Adenocarcinoma.* J Thorac Oncol, 2020. **15**(12): p. 1844-1856.

3. Chen J., Yang H., Teo A. S. M., Amer L. B., Sherbaf F. G., Tan C. Q., et al., *Genomic landscape of lung adenocarcinoma in East Asians.* Nat Genet, 2020. **52**(2): p. 177-186.

4. Bolger A. M., Lohse M. and Usadel B., *Trimmomatic: a flexible trimmer for Illumina sequence data.* Bioinformatics, 2014. **30**(15): p. 2114-20.

5. Tate J. G., Bamford S., Jubb H. C., Sondka Z., Beare D. M., Bindal N., et al., *COSMIC: the Catalogue Of Somatic Mutations In Cancer.* Nucleic Acids Res, 2019. **47**(D1): p. D941-D947.

6. Shen R. and Seshan V. E., *FACETS: allele-specific copy number and clonal heterogeneity analysis tool for high-throughput DNA sequencing.* Nucleic Acids Res, 2016. **44**(16): p. e131.

7. Rausch T., Zichner T., Schlattl A., Stutz A. M., Benes V. and Korbel J. O., *DELLY: structural variant discovery by integrated paired-end and split-read analysis.* Bioinformatics, 2012. **28**(18): p. i333-i339.

8. Thorvaldsdottir H., Robinson J. T. and Mesirov J. P., *Integrative Genomics Viewer (IGV): high-performance genomics data visualization and exploration.* Brief Bioinform, 2013. **14**(2): p. 178-92.

9. Chalmers Z. R., Connelly C. F., Fabrizio D., Gay L., Ali S. M., Ennis R., et al., *Analysis of 100,000 human cancer genomes reveals the landscape of tumor mutational burden.* Genome Med, 2017. **9**(1): p. 34.

10. Hu C., Zhao L., Liu W., Fan S., Liu J., Liu Y., et al., *Genomic profiles and their associations with TMB, PD-L1 expression, and immune cell infiltration landscapes in synchronous multiple primary lung cancers.* J Immunother Cancer, 2021. **9**(12).

11. Zhou Q., Tao F., Qiu L., Chen H., Bao H., Wu X., et al., *Somatic Alteration Characteristics of Early-Onset Gastric Cancer.* J Oncol, 2022. **2022**: p. 1498053.

12. Alexandrov L. B., Nik-Zainal S., Wedge D. C., Aparicio S. A., Behjati S., Biankin A. V., et al., *Signatures of mutational processes in human cancer.* Nature, 2013. **500**(7463): p. 415-21.

13. Sun Y., Qin S., Wang S., Pang J., Ou Q., Liang W. and Zhong H., *Comprehensive genomic profiling of pulmonary spindle cell carcinoma using tissue and plasma samples: insights from a real-world cohort analysis.* J Pathol Clin Res, 2024. **10**(3): p. e12375.

14. Sacher A. G. and Gandhi L., *Biomarkers for the Clinical Use of PD-1/PD-L1 Inhibitors in Non-Small-Cell Lung Cancer: A Review.* JAMA Oncol, 2016. **2**(9): p. 1217-22.

| **Table S1. Univariate analysis for disease-free survival using the study cohort (N=226)** | | |
| --- | --- | --- |
| Covariate | HR (95% CI) | *P*-value |
| *Clinical features* | | |
| Sex (male vs. female) | 1.87 (0.96-3.63) | 0.064 |
| Age (≥ 60 years vs. < 60 years) | 1.6 (0.83-3.07) | 0.161 |
| Histology (ADC vs. non-ADC) | 0.43 (0.17-1.11) | 0.083 |
| Clinical stage (II-III vs. I) | 17.64 (8.77-35.47) | <0.001*** |
| Smoking (yes vs. no) | 1.63 (0.69-3.83) | 0.266 |
| PD-L1 (high vs. low) | 2.79 (1.39-5.6) | 0.004** |
| TMB (high vs. low) | 2.17 (0.94-4.99) | 0.069 |
| MRD (MRD+ vs. MRD-) | 21.24 (10.47-43.08) | <0.001*** |
| *Mutational features (variant vs. wildtype)* | | |
| *RB1* | 5.49 (2.58-11.7) | <0.001*** |
| *TP53* | 7.01 (3.37-14.56) | <0.001*** |
| *EGFR* | 0.49 (0.25-0.95) | 0.034* |
| *GNAS* | 2.22 (0.68-7.28) | 0.189 |
| *PKHD1* | 1.43 (0.34-5.98) | 0.623 |
| *ERBB2* | 0.73 (0.18-3.06) | 0.670 |
| *KRAS* | 0.84 (0.33-2.17) | 0.719 |
| *LRP1B* | 1.17 (0.41-3.31) | 0.767 |
| *MED12* | 0.84 (0.2-3.48) | 0.805 |
| *ALK* | 0.84 (0.2-3.52) | 0.816 |
| *PLCB4* | 1.03 (0.25-4.29) | 0.968 |
| *PIK3CA* | 1.01 (0.24-4.19) | 0.993 |
| *BRAF* | 0 (0-Inf) | 0.996 |
| Abbreviations: HR, hazard ratio; CI, confidence interval; ADC, adenocarcinoma; TMB, tumor mutation burden; PD-L1, programmed death ligand 1; MRD, molecular residue disease | | |

## **Supplementary Tables**

| **Table S2. Univariate analysis for overall survival using the study cohort (N=226)** | | |
| --- | --- | --- |
| Covariate | HR (95% CI) | *P*-value |
| *Clinical features* | | |
| Sex (male vs. female) | 2.01 (0.66-6.15) | 0.220 |
| Age (≥ 60 years vs. < 60 years) | 5.25 (1.45-19.1) | 0.012* |
| Histology (ADC vs. non-ADC) | 0.44 (0.1-1.98) | 0.282 |
| Clinical stage (II-III vs. I) | 41.41 (9.11-188.31) | <0.001*** |
| Smoking (yes vs. no) | 1.98 (0.51-7.67) | 0.321 |
| PD-L1 (high vs. low) | 1.86 (0.56-6.18) | 0.311 |
| TMB (high vs. low) | 3.77 (1.15-12.35) | 0.029* |
| MRD (MRD+ vs. MRD-) | 18.41 (5.61-60.4) | <0.001*** |
| *Mutational features (variant vs. wildtype)* | | |
| *TP53* | 13.41 (2.97-60.56) | 0.001*** |
| *RB1* | 6.11 (1.87-19.93) | 0.003** |
| *LRP1B* | 4.5 (1.38-14.67) | 0.013* |
| *PKHD1* | 4.3 (0.94-19.66) | 0.060 |
| *ALK* | 2.7 (0.6-12.23) | 0.196 |
| *EGFR* | 0.53 (0.18-1.58) | 0.255 |
| *GNAS* | 1.81 (0.23-14.05) | 0.570 |
| *PLCB4* | 1.52 (0.2-11.67) | 0.690 |
| *PIK3CA* | 1.35 (0.18-10.41) | 0.772 |
| *MED12* | 1.15 (0.15-8.83) | 0.895 |
| *ERBB2* | 1.11 (0.14-8.52) | 0.922 |
| *KRAS* | 0.98 (0.22-4.44) | 0.976 |
| *BRAF* | 0 (0-Inf) | 0.998 |
| Abbreviations: HR, hazard ratio; CI, confidence interval; ADC, adenocarcinoma; PD-L1, programmed death ligand 1; TMB, tumor mutation burden; MRD, molecular residue disease | | |

| **Table S3. Univariate analysis for disease-free survival and overall survival using Xuanwei patients (N=31)** | | | | |
| --- | --- | --- | --- | --- |
|  | DFS | | OS | |
| Feature | HR (95% CI) | *P*-value | HR (95% CI) | *P*-value |
| Sex (male vs. female) | 1.16 (0.26-5.22) | 0.85 | 0.49 (0.04-5.40) | 0.56 |
| Age (≥ 60 years vs. < 60 years) | 4.13 (0.75-22.81) | 0.10 | 3.5e+09 (0-Inf) | >0.99 |
| Clinical stage (II-III vs. I) | 9.0e+09 (0-Inf) | >0.99 | 9.9e+09 (0-Inf) | >0.99 |
| Smoking (yes vs. no) | 0.59 (0.07-5.33) | 0.64 | 0 (0-Inf) | >0.99 |
| TMB (high vs. low) | 0.90 (0.13-6.40) | 0.92 | 0.86 (0.05-13.73) | 0.91 |
| PD-L1 (high vs. low) | 2.23 (0.37-13.47) | 0.38 | 2.81 (0.18-44.93) | 0.47 |
| Clinical stage (II-III vs. I) | 9.1e+09 (0-Inf) | >0.99 | 9.9e+09 (0-Inf) | >0.99 |
| MRD (MRD+ vs. MRD-) | 35.27 (4.02-309.57) | <0.001*** | 9.9e+09 (0-Inf) | >0.99 |
| *RB1* variants | 4.26 (0.76-23.77) | 0.10 | 3.57 (0.32-39.47) | 0.30 |
| *TP53* variants | 3.59 (0.69-18.67) | 0.13 | 1.9e+09 (0-Inf) | >0.99 |
| *EGFR* variants | 0.44 (0.1-1.96) | 0.28 | 0.31 (0.03-3.41) | 0.34 |
| *LRP1B* variants | 0.43 (0.05-3.57) | 0.43 | 1.36 (0.12-15.02) | 0.80 |
| *ALK* variants | 4.37 (0.5-37.88) | 0.18 | 11.07 (0.98-125.48) | 0.05 |
| *BRAF* variants | 0 (0-Inf) | >0.99 | 0 (0-Inf) | >0.99 |
| *ERBB2* variants | 0 (0-Inf) | >0.99 | 0 (0-Inf) | >0.99 |
| *GNAS* variants | 0.96 (0.11-8.43) | 0.97 | 3.57 (0.32-39.47) | 0.30 |
| *KRAS* variants | 1.31 (0.26-6.49) | 0.74 | 2.45 (0.22-27.02) | 0.47 |
| *MED12* variants | 0.53 (0.06-4.42) | 0.56 | 1.73 (0.16-19.06) | 0.66 |
| *PIK3CA* variants | 0 (0-Inf) | >0.99 | 0 (0-Inf) | >0.99 |
| *PKHD1* variants | 1.13 (0.13-9.65) | 0.91 | 2.93 (0.27-32.43) | 0.38 |
| *PLCB4* variants | 0 (0-Inf) | >0.99 | 0 (0-Inf) | >0.99 |

| **Table S4. The 10 mutational signature groups used for signature analysis** | |
| --- | --- |
| Mutation Signature | Description |
| Age | Signature 1 |
| APOBEC | Signature 2, Signature 13 |
| BRCA | Signature 3 |
| Smoking | Signature 4 |
| MMRdeficiency | Signature 6, Signature 15, Signature 20, Signature 26 |
| Ultraviolet | Signature 7 |
| Immunoglobulin | Signature 9 |
| POLE | Signature 10 |
| Temozolomide | Signature 11 |
| Others | 1- Age - APOBEC - BRCA - Smoking - MMRdeficiency - Ultraviolet - Immunoglobulin - POLE - Temozolomide |

## **Supplementary Figures**


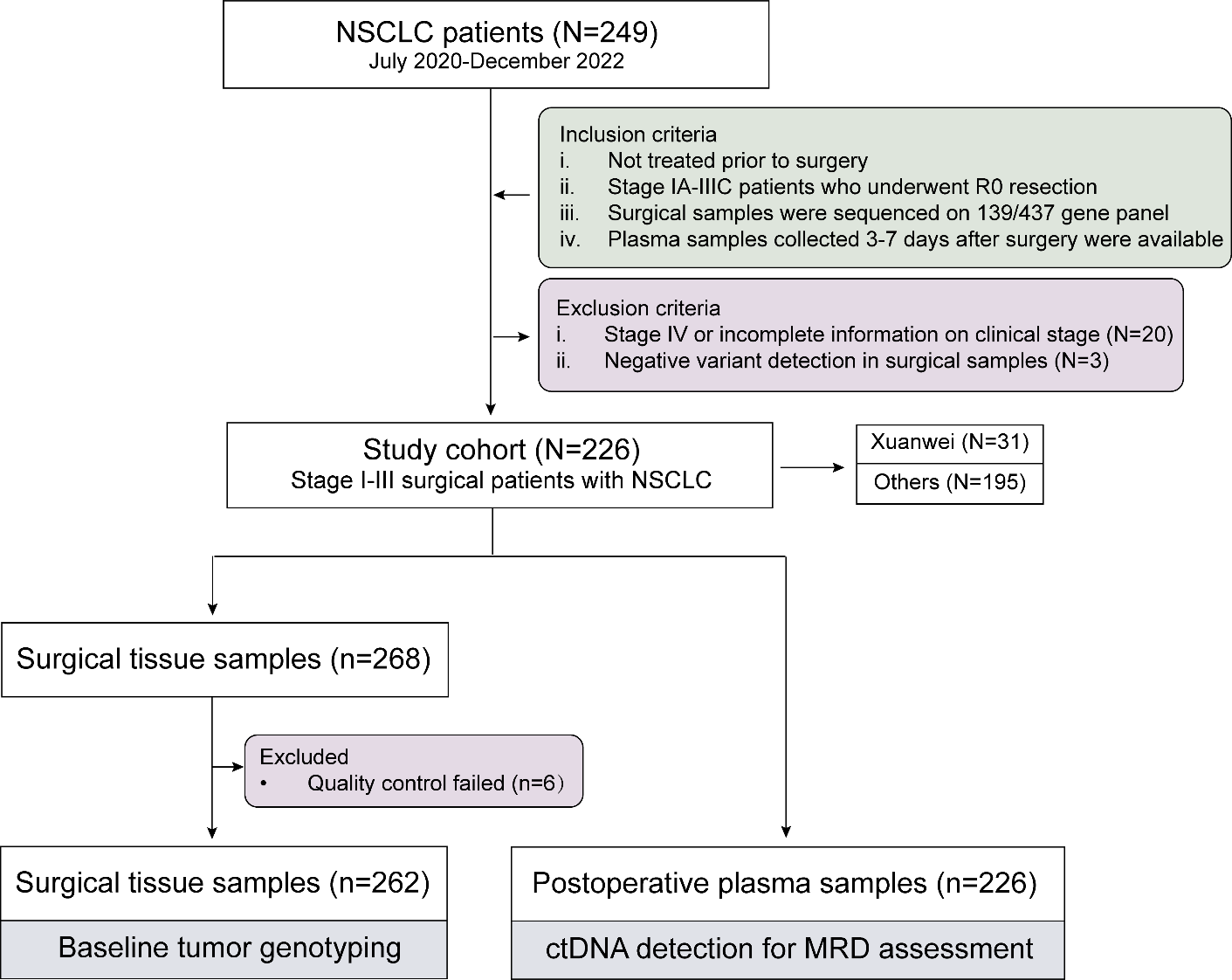


### **Figure S1. Overview of study design**

A total of 226 patients with histologically confirmed stage I-III NSCLC who underwent curative-intent surgery between July 2020 and December 2022 were included in this retrospective study. After excluding samples that failed quality control, a total of 262 primary tumors were further analyzed for baseline genomic profiling. Plasma samples collected 3-7 days post-surgery were analyzed using the identical platform as matched tumors for circulating tumor DNA (ctDNA) detection and molecular residual disease (MRD) assessment.


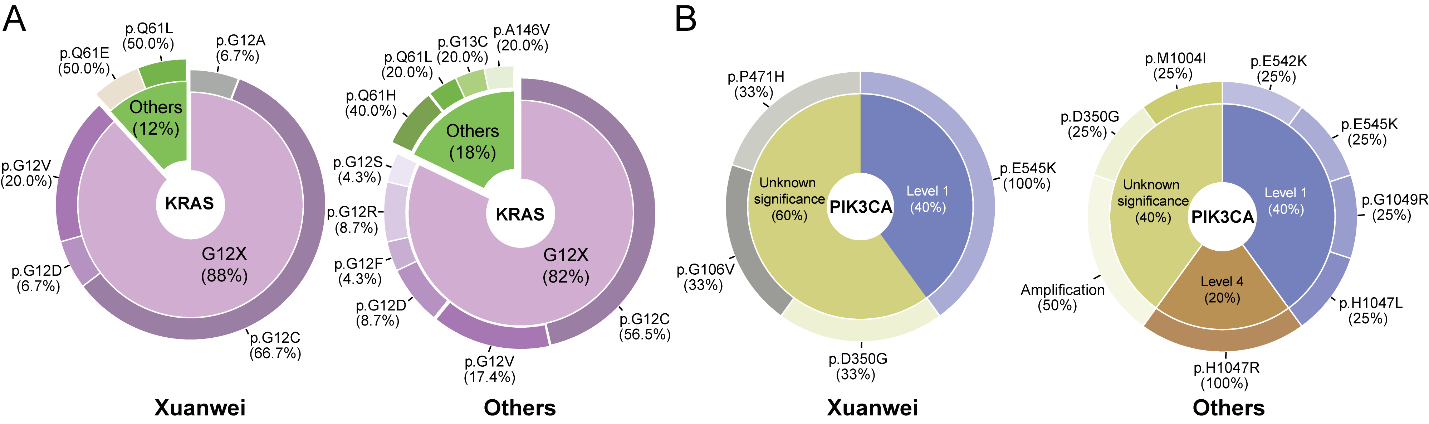


### **Figure S2. Distribution of *KRAS* and *PIK3CA* variants in patients from different regions**

(**A**) Type and proportion of *KRAS* mutations in patients from Xuanwei or other regions, categorized by the presence of G12X mutations. (**B**) Type and proportion of *PIK3CA* variants in patients from Xuanwei or other regions, categorized based on the OncoKB level of evidence for clinical actionability.


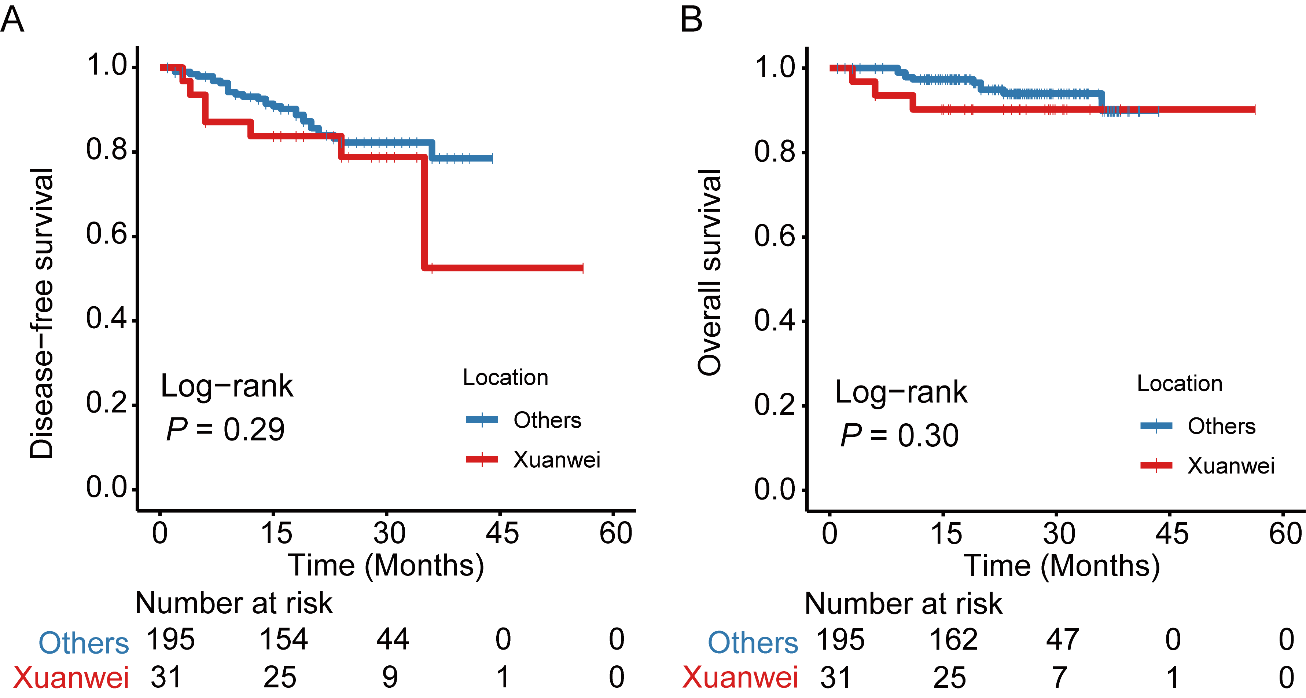


### **Figure S3. Survival analysis of patients associated with regional lung cancer risks.**

(**A**) Kaplan–Meier analysis of disease-free survival stratified by patient’s geographical location. (**B**) Kaplan–Meier analysis of overall survival of patients from Xuanwei and others.


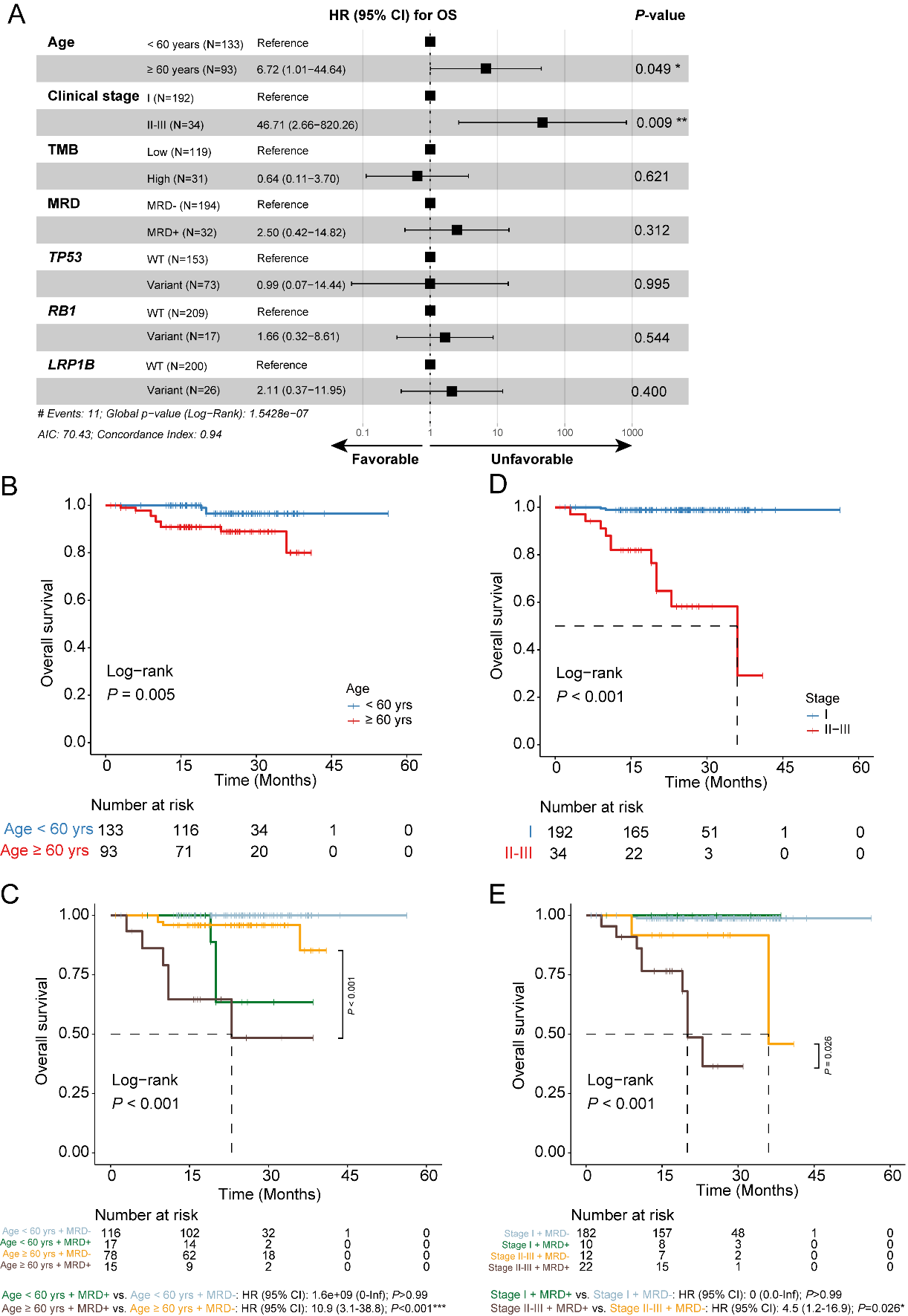


### **Figure S4. Detectable MRD associated with worse overall survival in surgical patients**

(**A**) Forest plot showing the multivariate analysis of hazard ratios (HR) with 95% confidence intervals (CI) for overall survival (OS). (**B, C**) Kaplan–Meier analysis of OS stratified by age or in combination with postoperative molecular residual disease (MRD) status. (**D, E**) Kaplan–Meier analysis of OS stratified by clinical stage or in combination with postoperative MRD status.
